# 

**Published:** 1932

**Authors:** 


### The Medical Library of the University of Bristol,

WITH WHICH IS INCORPORATED

**The Library of the Bristol Medico-Chirurgical Society.**

---

*The following donations have been received since the publication of the list in June,* 1932.

September, 1932.

| | | |
|---|---|---|
| American Otological Society (1) | .. .. | 1 volume. |
| Dental Board of the United Kingdom (2) | .. | 1 ,, |
| Michell Clarke Fund (3) .. .. .. | .. | 3 volumes. |
| Dr. M. Nierenstein (4) .. .. .. | .. | 1 volume. |
| Dr. J. A. Nixon (5) .. .. .. | .. | 1 ,, |
| University of Otago Medical School (6) | .. | 1 ,, |
| Dame Mary Monica Wills (7) .. .. | .. | 2 volumes. |

Unbound periodicals have also been received from Professor E. W. Hey Groves.

### THE ONE HUNDRED AND FORTY-FIRST LIST OF BOOKS.

The figures in round brackets refer to the figures after the names of the donors. The books to which no such figures are attached have either been bought from the Library Fund or received through the *Journal.*

| | | |
|---|---|---|
| **Alexander, W.** .. .. | *Plain and Easy Directions for use of Harrowgate Waters* .. .. .. .. .. .. .. .. .. .. | 1773 |
| *Australian and New Zealand Pharmaceutical Formulary* .. 5th Ed. | | 1930 |
| **Bailey, H., and Love, R. J. M.** | *Short Practice of Surgery,* Vol. I .. | 1932 |
| *Certain Necessary Directions . . . Cure of Plague . . . Prevention of Infection* . . . .. .. .. .. .. .. .. .. .. .. .. .. .. | | 1636 |
| **Conybeare, J. J.** .. | *Textbook of Medicine* .. .. .. 2nd Ed. | 1932 |
| **Cummings, B. F.** .. | *The Bed Bug* .. .. .. .. .. 3rd Ed. | 1932 |
| *Dental Board of the United Kingdom's Four Lectures on Practical Points Connected with Dental Mechanics* .. .. .. .. .. .. (2) | | |
| **Du Clos** .. .. .. | *Observations on the Mineral Waters of France* | 1684 |
| **Heald, C. B.** .. .. | *Injuries and Sport* .. .. .. .. .. .. .. | 1931 |
| **Hoffman, F.** .. .. | *New Experiments and Observations upon Mineral Waters* .. .. .. .. .. 2nd Ed. | 1743 |
| **Hunter, A.** .. .. | *The Waters of Harrogate* .. .. 5th Ed. | 1838 |
| **Needham, O. M.** .. | *The Bio-chemistry of Muscle* .. .. (4) | 1932 |

| | | | |
|---|---|---|---|
| | *Nouveau Traité de Médecine.* Vols. 2, 3 and 18 .. .. .. | (3) | 1928 |
| | *Spadacrene Anglica ; or the English Spaw* .. .. .. .. .. .. .. | | |
| **Walker, J.** .. .. .. | *Dissertatio Chemica inauguralis de aqua sulphurea Harrowgatensi* .. .. .. .. .. | | 1770 |
| **Wells, A. G.** .. .. | *The Discharging Ear* .. .. .. .. .. .. | | 1932 |

TRANSACTIONS, REPORTS, JOURNALS, ETC.

| | | |
|---|---|---|
| American Otological Society, Transactions. XXI .. .. .. | (1) | 1931 |
| Bath and Wells Diocesan Directory and Almanack .. .. .. .. | | 1921 |
| Burdett's Hospitals and Charities .. .. .. .. .. .. .. .. | (7) | 1916 |
| Collected Papers of the Mayo Clinic. XXIII (1931).. .. .. .. | | 1932 |
| College of Physicians of Philadelphia, Transactions. Vol. 53 .. .. | | 1931 |
| Henry Phipps Institute, University of Pennsylvania. 23rd Report.. | | 1931 |
| Researches published from the London Hospital during 1931 .. | (5) | |
| Surgical Clinics of North America. June, 1932 .. .. .. .. .. | | |
| University of Otago Medical School, Proceedings. No. 9 .. .. | | 1932 |

### Local Medical Notes

EXAMINATION RESULTS.

**University of Bristol.**—M.B., Ch.B. *Final Examination :* H. F. M. Finzel (Second Class Honours).

**Conjoint Board.**—M.R.C.S., L.R.C.P. *Pass. In Chemistry :* P. W. J. Parkes. *In Elementary Biology :* J. G. Jones. *In Anatomy and Physiology :* Ursula W. Wood. *In Anatomy :* T. H. Taylor. *In Medicine :* D. P. Dewe. *In Midwifery :* H. E. Pearse, F. E. Fletcher.* *In Pathology and Midwifery :* R. L. Marks. *In Midwifery :* Edith K. P. Harris. *In Pathology :* A. J. Board, G. L. Foss, E. O. Walker. *In Surgery :* C. N. Royal, Enid A. Taylour.*

L.D.S., R.C.S.—*Final Examination. Pass. Part I :* O. F. Harley, Margaret D. Hooper. *Part II :* R. E. Morgan.

**Society of Apothecaries.**—L.S.A. *Pass. In Anatomy :* K. P. Pauli.

**Long Fox Memorial Lecture.**—The 1932 Lecture will be delivered by Mr. A. J. Wright, F.R.C.S., on Tuesday, 25th October, at 5.30 p.m., in the Physiological Lecture Theatre of the University, the subject being "The Problem of Deafness."

* Qualified.

### Publications Received

From Edward Arnold & Co.:

*Heart Disease.* By Crighton Bramwell. Price 12s. 6d.

From the Trustees of the British Museum:

*The Bed Bug; its Habits and Life History.* Third Edition. By B. F. Cummings. Price 2d.

From Noel Douglas:

*On the Management of a Birth Control Centre.* Second Edition. By Evelyn Fuller. Price 1s. 6d.
*The Critical Age of Women.* By W. M. Gallichan. Price 4s. 6d.
*A Guide to Birth Control Literature.* By Norman E. Haines. Price 2s. 6d.

From Geoffrey Hadfield, Esq., M.D.:

*Recent Advances in Pathology.* By G. Hadfield, M.D., and L. P. Garrod, M.A., M.B., B.Ch. Price 15s.

From William Heinemann (Medical Books) Ltd.:

*The Francis Treatment of Asthma.* By A. Francis, M.B., B.Ch. Price 7s. 6d.

From H. K. Lewis & Co. Ltd.:

*A Shorter Surgery.* Third Edition. By R. J. McNeill Love, M.B., M.S. Price 16s.

From Oxford University Press:

*Handbook of the Vaccine Treatment of Chronic Rheumatic Diseases.* By F. Warren Crowe, D.M., B.Ch. Price 3s. 6d.

From John Wright & Sons Ltd.:

*British Journal of Surgery.* Vol. XX, No. 77. Price 12s. 6d.

From The Year Book Publishers, Chicago:

*Neurology and Psychiatry.* (Practical Medicine Series.) Edited by Peter Bassoe, M.D., and F. G. Ebaugh, A.B., M.D.

*Pediatrics.* (Practical Medicine Series.) Edited by Isaac A. and Arthur F. Abt, M.D.

*General Surgery.* (Practical Medicine Series.) Edited by Evarts A. Graham, A.B., M.D.

*Obstetrics and Gynæcology.* (Practical Medicine Series.) Edited by Joseph B. de Lee, A.M., M.D., and J. P. Greenhill, B.S., M.D.

*Dermatology, Syphilis and Urology.* (Practical Medicine Series.) Edited by F. Wise, M.D., M. B. Sulzberger, M.D., and J. H. Cunningham, M.D.

